# Testing the reliability of an AI-based large language model to extract ecological information from the scientific literature

**DOI:** 10.1038/s44185-024-00043-9

**Published:** 2024-05-16

**Authors:** Andrew V. Gougherty, Hannah L. Clipp

**Affiliations:** USDA Forest Service Northern Research Station, Delaware, OH USA

**Keywords:** Invasive species, Macroecology, Data mining

## Abstract

Artificial intelligence-based large language models (LLMs) have the potential to substantially improve the efficiency and scale of ecological research, but their propensity for delivering incorrect information raises significant concern about their usefulness in their current state. Here, we formally test how quickly and accurately an LLM performs in comparison to a human reviewer when tasked with extracting various types of ecological data from the scientific literature. We found the LLM was able to extract relevant data over 50 times faster than the reviewer and had very high accuracy (>90%) in extracting discrete and categorical data, but it performed poorly when extracting certain quantitative data. Our case study shows that LLMs offer great potential for generating large ecological databases at unprecedented speed and scale, but additional quality assurance steps are required to ensure data integrity.

## Introduction

The recent public release of multiple artificial intelligence (AI)-based language generating chatbots has garnered significant attention from both the public and scientific communities^1,2^. The ability of large language models (LLMs) to quickly process and synthesize large amounts of text and return a reasonable response to user queries has led to the suggestion that scientists could potentially begin to shift mundane, laborious, or time-consuming tasks to AI systems^3^. However, while it seems promising that LLMs can generate correct technical answers and seemingly reasonable responses, the tendency for LLMs to sometimes “hallucinate” or return objectively wrong information raises significant concern about whether LLMs, in their current (publicly available) state, can be relied upon to produce accurate results. Furthermore, biases in the training data can perpetuate errors that can be difficult to understand, given the “black box” nature of LLMs and the frequent lack of transparency in the data used for training^4^. As such, it is uncertain whether LLMs in their current form offer a useful tool for scientists that could improve productivity, efficiency, learning, and teaching or whether LLMs should be avoided as a research tool due to imprecision and unreliability. With AI being increasingly used in ecological studies, and the possibility of AI-based systems generating novel, testable hypotheses and predictions^5,6^, there is a pressing need to characterize the ability of AI-based systems to interact with ecological data.

Here, we sought to formally test the ability of an LLM to extract ecological information from scientific reports and, in the process, generate a database that could undergo further analysis. We focused on scientific reports of plant pathogens occurring on new hosts or in new geographic regions (i.e., emerging infectious diseases [EIDs]). These reports provide a valuable real-world case study, as thousands of new disease reports are published annually in the scientific literature—indeed, there are entire journals dedicated to the topic (e.g., *New Disease Reports*, Online ISSN:2044-0588)—produced at a rate that would challenge any researcher to keep up-to-date on this rapidly expanding literature^7^. These reports also provide important information for understanding the spread of invasive species, which may harm ecosystems^8^, native communities^9^, and crop production^10^, and for informing future management and surveillance of invasive species. Based on the results of our case study, we identified the strengths and weaknesses of the LLM in extracting different types of data, and we conclude by commenting on the potential usefulness of LLMs in general as a research tool.

## Results and discussion

In total, data extraction via the LLM took approximately 5 min, while the reviewer took approximately 268 min to review the 100 reports, representing an over 50-fold difference. The LLM had a strong ability to accurately identify the pathogens, hosts, years, and countries described in the reports. Of the 103 pathogens described in the reports, 98.1% matched those identified by the reviewer (Kappa = 0.98, CI = 0.95–1.0). The only instances that were not exact matches were associated with the alder yellows pathogen. In this case, the reviewer correctly identified the pathogen as “Alder yellows phytoplasma,” while the LLM identified the pathogen as “Candidatus Phytoplasma alni.” While this pathogen name seems correct, it was not specifically mentioned in the report, nor were we able to find any mention of this specific pathogen name in the literature. Interestingly, this species name seems to follow the convention of other phytoplasmas (e.g., Candidatus *Phytoplasma ulmi*, Ca. *P*. *fraxini*). Host identities were also matched with high accuracy. Of the 132 hosts identified by the reviewer or LLM, 91.7% were exact matches (Kappa = 0.92, CI = 0.87–0.96). The greatest errors were due to omission—that is, instances where the reviewer identified a host that the LLM did not. These were exclusively cases where multiple hosts were listed in the report, but the LLM failed to identify all of the hosts. A similar trend was found for the year during which EIDs were observed. Generally, the reviewer and LLM returned identical years, with an overall accuracy of 72.1%, which consisted of 106 “true positive” matches, 11 “false positive” cases, 14 mismatching values, 15 errors of omission [due primarily to the LLM missing hosts or locations identified by the reviewer], and 1 error of commission out of 147 total cases. Mismatched values often occurred when the EIDs were observed across a range of multiple years. Countries were identified with the highest accuracy rate—effectively, all countries identified by the LLM were exact matches (100%) to those identified by the reviewer (Kappa = 1.0).

**Fig. 1:**
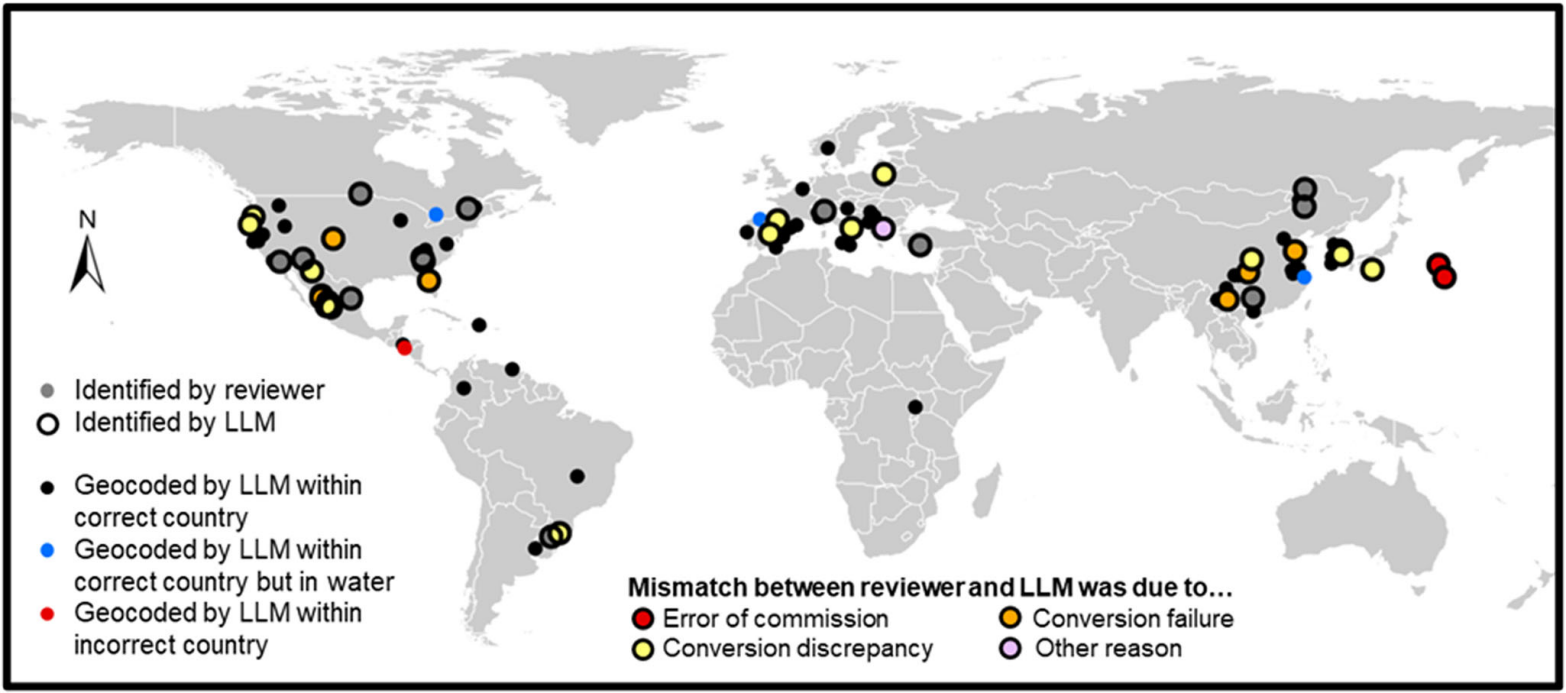
Geographic locations of emerging infectious tree diseases from 100 reports. Gray points (*N* = 44) are unique locations provided explicitly in the disease reports and identified by the human reviewer, and all other points (*N* = 110) are those identified by the large language model (LLM). In many cases, geographic coordinates were not provided in the disease reports, but the LLM automatically geocoded 70 unique locations, with high accuracy (98.6%) for placement in the correct country (black points) but uncertain precision (e.g., 3 sets of coordinates were located in bodies of water [see blue points]). Approximately 34.0% of the coordinates provided in the reports were precise matches (aside from negligible rounding issues) between values identified by both the reviewer and LLM (i.e., gray points with black border). Mismatches were due to small discrepancies in converting to decimal degrees (*N* = 16; yellow points), complete failure to correctly convert to decimal degrees (*N* = 8; orange points), errors of omission (*N* = 4; gray points with no border), errors of commission (*N* = 2; red points; note that these coordinates also failed to correctly convert to decimal degrees and resulted in the 2 points east of Japan, which should have been located in Australia), and conflation of 2 different sets of coordinates as a single set of coordinates (*N* = 1; purple point).

When latitude/longitude coordinates were supplied in the report (*N* = 34 out of the 100 total reports, comprising 44 unique locations where diseases were first recorded; Fig. 1), they tended to be similar between the LLM and reviewer, but the LLM frequently struggled with converting given coordinates to decimal degrees. There were 46 total unique locations identified by both the reviewer and LLM from the reports, and 34.0% of the latitude/longitude coordinate values were an exact match (aside from negligible rounding issues that occurred when converting to decimal degrees and resulted in mean absolute differences in latitude and longitude of 0.0002 and 0.0004, respectively). For 16 locations, minor mismatches arose from seemingly random discrepancies in converting to decimal degrees, which resulted in mean absolute differences in latitude and longitude of 0.1369 and 0.0022, respectively. For 8 locations, the LLM completely failed to convert correctly to decimal degrees, returning latitude and longitude values that had mean absolute differences of 0.1733 and 0.1097, respectively. The remaining mismatches were due to errors of omission (*N* = 4), errors of commission (*N* = 2), and a case where 2 different locations were conflated and treated as a single location. Excluding those latter 7 cases, absolute differences ranged from 0–1.8383 (mean = 0.1052) for latitude and from 0–0.2800 (mean = 0.0270) for longitude.

It is worth noting that there were 2 cases where the reviewer was able to interpret location-related information that the LLM could not. The first instances were 2 errors of commission by the LLM in returning latitude/longitude coordinates that were not identified by the reviewer; both were from a single report that contained 2 sets of coordinates for surveys that had been conducted to monitor for tree diseases but were not the actual coordinates of the first records of the disease. The reviewer was able to distinguish the context and indicated NA for the latitude/longitude coordinates, as they were not provided, but the LLM returned the 2 sets of tree survey coordinates. Thus, in this case, the LLM appeared to simply return any coordinates that were explicitly included in a report, even though they did not correspond to the requested location of a first disease record. The second instance was where the authors of the report apparently recorded the wrong longitude coordinate, resulting in a location that was far outside of the country where the disease was observed. The reviewer was able to correctly identify this inconsistency, but the LLM did not. While this is not entirely surprising, it verifies the LLM does not confirm the data it returns are internally consistent and, therefore, may be unlikely to identify errors in the inputted data without additional instruction.

Surprisingly, even when the report did not explicitly supply latitude/longitude coordinates, the LLM geocoded the locations (Fig. 1), producing coordinates for 70 unique locations with high accuracy (98.6%) for placement within the correct country. We were not expecting this behavior, but automatically geocoding the locations adds significant value to the extracted data, as climatic, land use and other environmental data could then be extracted for those locations. However, the LLM’s method of determining the geocoded location when it is not provided in the report is unknown, and we were unable to determine the reason for the single set of coordinates that were located within the incorrect country (~5 km from the border of the correct country). Furthermore, the precision of the geocoded locations is uncertain, particularly as 3 sets of coordinates were situated within bodies of water (Fig. 1), ranging 40 m to 5.2 km from the nearest shoreline.

The least accurate data extracted by the LLM were for pathogen incidence, which had an overall accuracy of 23.8% (consisting of 25 “true positive” matches, 10 “true negative” matches, 95 “false positive” cases, 1 mismatching value, 15 errors of omission [due primarily to the LLM missing hosts or locations identified by the reviewer], and 1 error of commission out of a total of 147 cases). Although the prompt specifically stated that NAs should be used when the data were not available in the report, the LLM assigned 53 of the 100 total reports with no incidence data as 100% incidence, whereas the reviewer returned an NA, and it was not clear how the LLM reached this answer. Of the cases where the reviewer identified an incidence value, the LLM frequently extracted the same value (96.2% matches, *N* = 25/26), which seems to indicate that the LLM can extract numeric data, but the frequency of “false positive” cases was concerning.

### Implications, limitations, and future directions

The workflow we utilized to automate data extraction from scientific reports highlights the potential for LLMs to rapidly generate large databases with relatively high accuracy, opening the potential for researchers to address new questions at a scale that was previously not possible. That said, there are numerous caveats to the approach we present, and to LLMs generally, that should be noted. First, the reports from which we extracted data were known *a priori* to have relevant pathogen, host, and geographic information. This use of generative AI is likely “safer” than some others, as we only asked the LLM to extract data we suspected were in the reports rather than find new data or generate text that was not already in the report. Further, the reports were short in length and relatively data-dense, so while the LLM was usually able to identify relevant data, it is yet unclear how well it would interact with longer text sources (e.g., journal articles with thousands of words). Further, the data we were extracting were relatively simplistic (e.g., pest/host scientific names, country names), which likely facilitated high accuracy rates. Our results are largely consistent with ref. ^11^, who suggested LLMs respond best to simple, straightforward tasks that do not require multiple sequential steps. Despite its simplicity, however, we note this type of information is valuable for tracking pathogens, pests, and invasive species in new areas and could be useful for both automating surveillance and identifying high-risk pathogens and pests before they arrive in a new region.

The LLM’s ability to distinguish between pathogen and host names was particularly useful as it indicates the LLM is not simply searching for formatting clues of scientific names (e.g., italics). Rather, it implies the pathogen and host names are evident from the context of the text and/or that possibly some of these scientific names occur in the data used to train the LLM. Interestingly, several of the pathogens in the reports were parasitic plants, which the LLM correctly identified as the pathogen—again suggesting an ability to distinguish between the antagonistic species and host species. However, the LLM did not consistently extract the quantitative data correctly. The tendency to assign 100% incidence when no incidence data were provided was somewhat disconcerting, and it was unclear why this occurred. If the general workflow from our case study were to be used to generate quantitative datasets, numerous quality assurance steps would need to be taken to ensure reasonable accuracy before further analysis. The fast pace of development of publicly available LLMs could overcome these issues in the near future, and formal comparison of multiple LLMs could clarify differences in their ability to interact with ecological data.

Despite the relatively high accuracy of the data extracted, the workflow could yet be improved. For instance, although the “off-the-shelf” LLM from our case study produced acceptable results for much of the data, fine-tuning an LLM could help improve the accuracy of the incidence or other quantitative data^12^. An LLM trained in ecological text could be particularly advantageous for more complex types of data, such as when effect sizes are needed for a meta-analysis. However, even the ability to reliably extract discrete/categorical data can prove tremendously valuable as these types of data could be used to identify novel ecological associations, biological introductions/invasions, and interactions between species and their environment. The ability of LLMs to interpret an expanding range of languages can also help overcome biases that may emerge when focusing solely on the English-language scientific literature^13,14^. However, the growing number and capabilities of LLMs, along with their associated data processing requirements, should warrant a certain amount of reflection on their use as cumulative environmental costs have yet to be fully realized^15^.

## Methods

### Source text

We used reports from a recent study on the global accumulation of emerging infectious tree diseases^7^. EIDs are generally defined as diseases occurring in a new geographic region, on a new host, or recently increasing in impact^16^. For plant species, EIDs are frequently documented in the literature as “First reports”, in which authors describe the conditions where the pathogen was detected and the methodological approaches used to identify the pathogen. These reports are frequently short in length, similar to the word count of a typical abstract (e.g., current guidelines for *Plant Disease*, the source of the disease reports used in our case study, state that reports should be ≤2985 characters). Because these reports are known to contain new and important ecological information, they offer a unique opportunity to test an LLM’s ability to extract relevant ecological information from the scientific literature. We used the first 100 reports from ref. ^7^, which represent unique hosts and pathogens reported in new regions.

### LLM data extraction

For our case study testing the ability of an LLM to extract various types of ecological data from the scientific literature, we elected to use the publicly available *text-bison-001* generative text model from Google. As a generative text model, *text-bison-001* is designed to return only the relevant text requested, which can then be parsed to a table, without the superfluous conversational text that would accompany responses by a chat model (e.g., OpenAI’s ChatGPT, Google’s Bard). In addition, the *text-bison-001* model can be accessed freely from an API, which allows for an entirely scripted workflow that bypasses the need to manually copy and paste data to and from a web browser.

We prompted the LLM to extract multiple pieces of information from the disease reports, including the scientific names of the pathogen and hosts described in the report, the incidence of the pathogen (i.e., percent of hosts affected by the pathogen), and when (i.e., year) and where (i.e., country) the pathogen was detected. The development of the prompt required some iterative experimentation to ensure that the requested data were returned accurately and consistently. In initial testing, for instance, we found that the LLM sometimes returned geographic coordinates as degrees–minutes–seconds or returned common names when scientific names were available. We found that explicitly stating the desired format for these variables increased the consistency of data extraction. Furthermore, because the data table was returned as a single text string, we realized that we needed to request that columns be delimited by a vertical bar (as opposed to a comma), because locations occasionally included commas, and this specification was necessary to properly delimit the table. As part of our workflow, the title and text of each report were appended to a text prompt, which described the data we wished to extract and the desired format of the response. The prompt read:

“The following is an abstract describing a plant pathogen on a new host or in a new geographic area.

I’d like to know (i) what is the scientific name of the pathogen?

(ii) what is the scientific name of the host?

(iii) what percentage of hosts were infected by the pathogen?

(iv) what year was the pathogen sampled?

(v) where was the pathogen observed?

(vi) what country was the pathogen observed in? and

(vii) what are the latitude/longitude, in decimal degrees, of the location where the pathogen was observed.

For the coordinates, don’t include letters to indicate the cardinal directions, but use negative numbers to indicate west and south.

It is very important that the latitude and longitude be returned in decimal degrees.

If there are multiple pathogens, hosts, or locations, include each as a separate row.

If any of the information is not included in the abstract, use NA.

Use a vertical bar | to delimit the table. Use the column names: ‘Pathogen’ [scientific name of pathogen], ‘Host’ [scientific name of host], ‘Percentage’ [percent of hosts infected], ‘Year’ [year pathogen was sampled], ‘Location’ [location where the pathogen was observed], ‘Country’ [country where pathogen was observed], ‘Latitude’ [latitude in decimal degrees], ‘Longitude’ [longitude in decimal degrees].

Always use scientific names when possible. Do not summarize the abstract. Return only a table. Here is the title, followed by the abstract:”

We interacted with the LLM through Google’s developer API, which was accessed with the httr package^17^ in the R statistical program^18^. The data from each report were returned as a single text string, with rows delimited with a newline designator (\n) and columns delimited with a vertical bar (|). The workflow was entirely scripted, and the table with the relevant responses was saved as an Excel file. The entire script to interact with the LLM is available at 10.6084/m9.figshare.24646302.

In initial testing, we found the LLM occasionally flagged the prompt and reports as being “derogatory” or “toxic” and would not return a response. It was not immediately clear why these particular reports might be considered derogatory or toxic. We were able to adjust the thresholds for the allowable derogatory/toxic content level in the response, which fixed the issue for the problematic reports. Of the 100 reports tested, 87 returned a result without adjusting the allowable derogatory/toxicity level, and 13 returned a result only after adjusting the allowable derogatory/toxicity level. Furthermore, to improve the repeatability of the responses, we set the “temperature” of the response to zero. Temperature controls the degree of creativity and stochasticity in the response. Setting the temperature to zero made the responses more deterministic, as the model always selected the highest probability response (see the API guide: https://cloud.google.com/vertex-ai/docs/generative-ai/model-reference/text).

### Validation

We tested how well the data extracted by the LLM compared to those extracted by an independent human reviewer (H. L. Clipp; hereafter, reviewer), who had not worked with these data previously. For the discrete variables (i.e., the identity of the pathogen, host, country, and year of sampling), we calculated 2 validation statistics: (i) an overall accuracy metric that was calculated as the number of exact matches between the LLM and reviewer divided by the total number of unique returns by the LLM and reviewer, and (ii) Cohen’s Kappa using the psych package in the R statistical program^19^. For the quantitative variables (i.e., latitude/longitude, incidence), we calculated LLM accuracy as the percentage of unique values that matched between those identified by both the LLM and reviewer, and we tallied the reasons for discrepancies (e.g., errors of commission/omission). We additionally calculated the absolute differences between unique latitude and longitude values returned by the LLM and reviewer. Any discrepancies between the reviewer and the LLM were assessed by the first author (A. V. Gougherty) to confirm the reviewer had extracted the correct information. We allowed some flexibility when the reviewer and LLM had minor disagreements. For instance, when hosts were identified to the subspecific level, we considered it a match whether or not the reviewer or LLM included “subsp.”, “ssp”, or no specific designation for the sub-specific epithet. Similarly, when the host was identified only to the genus level, we allowed the inclusion, or not, of “sp.” or “spp.” as a species identifier.

## Data Availability

The text of the disease reports and script to interact with the LLM is available at 10.6084/m9.figshare.24646302.
